# Dietary self-regulation levels among adults in Shanghai: cross-sectional associations with e-health literacy, perceived stress, and rest intolerance

**DOI:** 10.3389/fpubh.2026.1928922

**Published:** 2026-10-27

**Authors:** Fang Tian, Lin Du, Hao Yang, Yue Zhang

**Affiliations:** 1 Department of Critical Care Medicine, General Hospital of Ningxia Medical University, Yinchuan, China; 2 Department of Dermatology, Shanghai Ninth People’s Hospital, Shanghai Jiao Tong University School of Medicine, Shanghai, China; 3 Cardiac Center, General Hospital of Ningxia Medical University, Yinchuan, China

**Keywords:** dietary self-regulation, e-health literacy, indirect association analysis, latent profile analysis, perceived stress, rest intolerance

## Abstract

**Background:**

Dietary self-regulation supports healthy eating, but its cross-sectional associations with e-health literacy, perceived stress, and rest intolerance remain unclear. This study characterized dietary self-regulation levels among adults in Shanghai and estimated direct and indirect associations among these variables.

**Methods:**

A cross-sectional survey of 1,011 permanent adult residents of Shanghai was conducted from July to September 2024. Validated questionnaires assessed e-health literacy, dietary self-regulation, perceived stress, and rest intolerance. Associations were examined using Spearman correlations, exploratory latent profile analysis, and a prespecified serial indirect-association model (PROCESS Model 6; 5,000 bootstrap resamples) adjusted for gender, age, education, employment, residence, and income.

**Results:**

The selected three-class solution primarily distinguished low, moderate, and high overall dietary self-regulation, with largely parallel item-response patterns rather than qualitatively distinct subtypes. E-health literacy was positively associated with dietary self-regulation and rest intolerance and negatively associated with perceived stress; perceived stress was negatively associated with dietary self-regulation, whereas rest intolerance was positively associated with it. The total association between e-health literacy and dietary self-regulation was 0.615 (95% CI, 0.564–0.666); the association conditional on perceived stress and rest intolerance was 0.211 (95% CI, 0.147–0.274), and the total indirect association was 0.404 (95% CI, 0.344–0.466). The serial indirect association was small and negative (−0.015; 95% CI, −0.031 to −0.001), and the perceived stress-rest intolerance coefficient reversed direction after adjustment.

**Conclusion:**

The variables were interrelated in the prespecified cross-sectional model, but the small serial estimate and directional instability do not support a clear sequential psychological mechanism. Because all variables were measured at one time point, temporal ordering and causal mediation cannot be established, and reverse causality remains possible. These findings are hypothesis-generating and require longitudinal or experimental confirmation.

## 1 Introduction

### 1.1 Research background

Dietary behavior, encompassing conscious food selection, portion control, and nutritional planning, constitutes a fundamental component of non-communicable disease prevention. The World Health Organization emphasizes that healthy diets help prevent malnutrition and non-communicable diseases, including diabetes, cardiovascular disease, and cancer (1). In China, the ongoing nutrition transition and rapid increase in obesity have intensified the public-health burden of diet-related non-communicable disease (2). At the same time, highly processed foods rich in unhealthy fats, free sugars, and sodium create a more challenging dietary environment for self-regulation. Current research primarily explains dietary modification behaviors from cognitive, motivational, and self-regulatory perspectives: attitudes, subjective norms, perceived behavioral control, and intrinsic motivation collectively influence healthy eating intentions. It emphasizes that self-efficacy, stage-appropriate adaptation, and readiness for behavioral change can improve dietary behaviors, while risk perception, benefit perception, perceived barriers, and self-efficacy predict positive dietary change (3–5). Furthermore, models such as the COM-B framework, the Health Action Process Approach, and socio-ecological models are used to explain capability, opportunity, motivation, action planning, and multilevel environmental barriers to dietary modification. These models underscore that healthy eating is shaped not only by individual volition but also by external determinants, including family support, food affordability and accessibility, community environments, and policy-level factors (6–8). The intention-behavior gap is moderated by psychological wellbeing and dysfunctional eating patterns, underscoring the need to consider these regulatory processes (9).

However, despite increasing attention to dietary regulation, existing research has not fully explained why individuals with health-related knowledge or intentions may still experience difficulties in regulating their eating behaviors. One possible limitation is that many studies have primarily emphasized rational cognition and personal responsibility, while giving less consideration to psychological strain and context-related emotional experiences. In the digital health context, eHealth literacy may influence how individuals obtain and use health information related to diet, but its association with dietary regulation behaviors remains underexplored. At the same time, rest intolerance and perceived stress may be important psychological correlates of dietary regulation, as they reflect individuals’ emotional burden and stress experience in daily life. However, limited research has examined these variables simultaneously or explored their possible mediating roles. To address this gap, the present study investigates the relationships among eHealth literacy, rest-related shame, perceived stress, and dietary regulation behaviors.

### 1.2 The role of e-health literacy

E-health literacy denotes the capacity to seek, understand, evaluate, and apply online health information to address health concerns (10). The widespread adoption of the Internet and mobile devices has expanded access to health information, making e-health literacy an important correlate of health behavior. Higher e-health literacy has been associated with health-promoting behaviors, including dietary regulation, and may operate through health knowledge and self-efficacy (11). Interactive health literacy and e-health literacy have also been associated with healthier dietary patterns, greater consumption of nutritious foods, and lower intake of unhealthy foods (12, 13). Broader health literacy may support the self-regulation of eating behavior through physical activity and self-efficacy pathways (14). However, most prior studies have focused on students or specific demographic groups, and evidence from community residents remains limited.

### 1.3 The role of rest intolerance

In recent years, rest intolerance has attracted attention as an emerging psychological construct. It refers to negative affective and cognitive reactions—such as guilt, anxiety, self-blame, shame, or persistent task-related thoughts—when individuals rest, engage in leisure, or temporarily disengage from work or study (15, 16). Viewing leisure as wasteful may reduce its enjoyment and restorative value and is associated with poorer psychological wellbeing (17). In the Chinese cultural context, rest intolerance may be shaped by achievement orientation, social comparison, and an emphasis on sustained effort. The RIS-8 includes negative feelings, obsessive thinking, social comparison, and cognitive bias dimensions (15). Dietary self-regulation involves modulating food intake in relation to health goals, hunger-satiety signals, and emotional states and includes self-control, emotion regulation, and goal maintenance (18, 19). Negative psychological states, including stress, anxiety, depression, and shame, are associated with emotional eating, loss-of-control eating, and maladaptive restrictive eating (20–22). Rest intolerance may therefore be associated with dietary self-regulation, but the direction of this association is uncertain. Direct evidence remains limited, and shared or unmeasured characteristics may contribute to any observed relationship. We therefore examined rest intolerance as a prespecified statistical correlate.

### 1.4 The role of perceived stress

Perceived stress refers to the extent to which individuals appraise life demands as unpredictable, uncontrollable, and overwhelming relative to their coping resources (23). It is an important psychological correlate of health-related behavior, including dietary regulation. Stress has been associated with greater intake of energy-dense, high-fat, or high-sugar foods and with weaker healthy food choices and dietary control through emotional arousal, reward sensitivity, and reduced self-control (22). Higher perceived stress has also been associated with emotional and uncontrolled eating, poorer adherence to healthy dietary patterns, and difficulty maintaining dietary change (24–26). However, existing studies have mainly focused on emotional eating, unhealthy food intake, or overall diet quality, with limited attention to multidimensional dietary regulation. The pattern and direction of associations between perceived stress and dietary self-regulation in community populations remain uncertain.

The proposed ordering of the mediators was guided by Lazarus and Folkman’s Transactional Model of Stress and Coping (27), which posits that individuals first appraise whether environmental demands exceed their available coping resources and subsequently develop cognitive, emotional, and behavioral responses. Within this framework, e-health literacy was conceptualized as a personal coping resource that may influence the appraisal of health-related demands, perceived stress as the proximal appraisal of an imbalance between demands and coping resources (23), and rest intolerance as a more specific cognitive-affective response pattern expressed when individuals attempt to disengage from ongoing demands and rest (15). Accordingly, perceived stress was positioned as the first mediator and rest intolerance as the second, with dietary self-regulation conceptualized as a downstream health behavior that may be associated with this appraisal-and-response process. This ordering is also consistent with evidence from a recent Chinese sample showing that higher perceived stress was independently associated with greater rest intolerance (16). Nevertheless, the proposed sequence represents a theory-informed statistical specification rather than an established temporal or causal pathway. Given the cross-sectional design, reverse ordering and reciprocal relationships remain plausible and cannot be excluded.

Previous studies have examined the association between e-health literacy and dietary self-regulation, but the joint cross-sectional associations involving perceived stress and rest intolerance remain unclear. This study therefore used exploratory latent profile analysis to assess whether dietary self-regulation responses reflected qualitatively distinct configurations or mainly differences in overall level. It also estimated direct and prespecified indirect associations among e-health literacy, perceived stress, rest intolerance, and dietary self-regulation in adults living in Shanghai. The following hypotheses were tested:

*H1*: E-health literacy is positively associated with dietary self-regulation.

*H2*: The association between e-health literacy and dietary self-regulation includes a specific indirect association involving perceived stress.

*H3*: The association between e-health literacy and dietary self-regulation includes a specific indirect association involving rest intolerance.

*H4*: The association between e-health literacy and dietary self-regulation includes a serial indirect association involving perceived stress followed by rest intolerance.

## 2 Methods

### 2.1 Research design

A quota-based convenience sampling approach was used within the cross-sectional Psychology and Behavior Investigation of Chinese Residents (PBICR) survey conducted from July 10 to September 15, 2024 (28). Eligible participants were aged ≥18 years, were Chinese nationals residing in China for at least 1 month, lived in Shanghai on a long-term basis, and were able to read and understand the questionnaire. Recruitment in Shanghai was guided by predefined age quotas that approximated the adult age distribution: 13.2% aged 18–25 years, 13.2% aged 25–30 years, 17.0% aged 31–40 years, 18.9% aged 41–50 years, 17.0% aged 51–60 years, 11.3% aged 61–70 years, and 9.4% aged ≥71 years. Although the planned sample size was approximately 1,000, 1,245 questionnaires were returned. After age-quota matching, 1,011 participants were included in the final analysis, while 234 questionnaires were not retained because some age strata exceeded their predefined quotas. Thus, 81.2% of the returned questionnaires were retained (Figure 1). Participants completed the questionnaire independently or with interviewer assistance.

**Figure 1:**
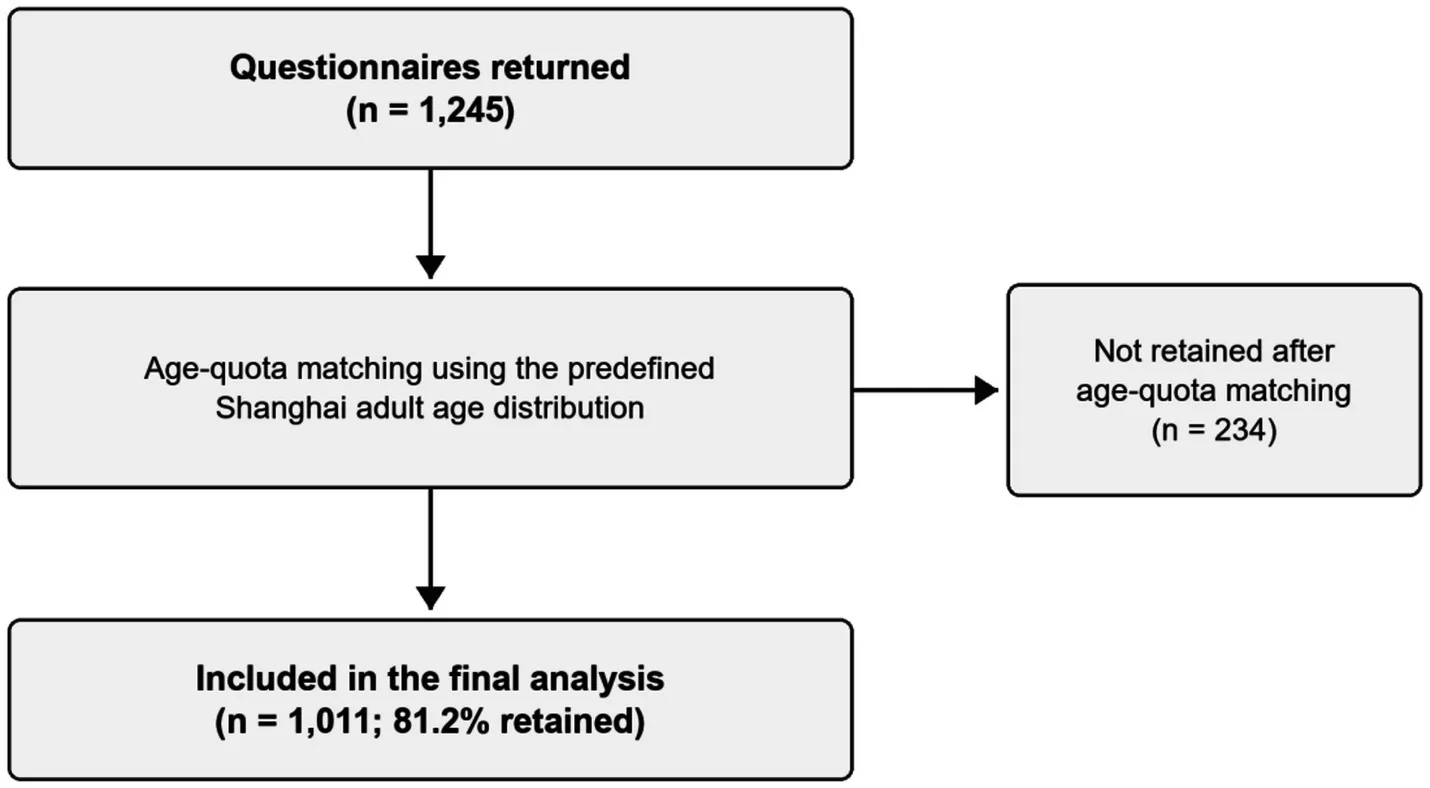
Participant flow from questionnaire return to the final age-quota-matched analytical sample.

Sample-size adequacy was evaluated according to recommendations for multivariable regression and indirect-effect analysis, for the most complex model with nine predictors, Green’s formula (N ≥ 50 + 8 m) indicated a minimum sample of 122 participants (29–31). The final sample of 1,011 exceeded this threshold and was considered adequate for bootstrap indirect-association analysis. For the exploratory latent profile analysis, adequacy was assessed based on model convergence, replication across random starts, entropy, profile size, and theoretical interpretability.

Green’s formula is best understood as a heuristic for the overall multivariable regression model rather than as a formal power calculation for latent profile analysis or the serial indirect-effect estimate. Consequently, the final sample of 1,011 was regarded as sufficient for the planned bootstrap estimation and exploratory LPA, with adequacy established by stable model convergence, replication across random starts, entropy, profile size, and interpretability, rather than as evidence of *a priori* statistical power for all analyses.

Ethical authorization and informed consent. This study was conducted in accordance with the ethical standards of the Declaration of Helsinki and received approval from the ethics committee of Shanghai Jiao Tong University (No. H202402371). Each participant provided written informed consent before participation and could withdraw from the study at any time. Participant privacy and data confidentiality were protected, and all information was handled anonymously. Survey data were used solely for statistical analysis.

### 2.2 Research instruments

#### 2.2.1 Covariates

Demographic and socioeconomic characteristics of participants were collected as covariates, including age, gender (female, male), region (urban, rural), education level (elementary school, junior high school, high school, bachelor’s degree, master’s degree or above), job (employed, student, retired, unemployed), monthly per capita household income (≤3,000, 3,000–5,000, 5,000–9,000, >9,000).

#### 2.2.2 Rest intolerance

Rest intolerance was assessed using the 8-item Rest Intolerance Scale (RIS-8) (15). The scale consists of four dimensions: negative feelings, obsessive thinking, social comparison, and cognitive bias, with two items in each dimension. Items are rated on a 5-point Likert scale ranging from “strongly disagree” (1) to “strongly agree” (5). Higher total scores indicate stronger negative reactions to rest, that is, greater rest intolerance; higher scores should not be interpreted as greater tolerance or adaptive self-control. The original validation study reported a Cronbach’s *α* of 0.89. In the present study, Cronbach’s *α* was 0.932.

#### 2.2.3 Dietary self-regulation

The present study employed the Self-regulation of Eating Behavior Questionnaire (SREBQ), as developed by Kliemann et al. (32). This instrument comprises five items, each rated on a 5-point Likert scale ranging from “Never” (1) to “Always” (5). Total scores range from 5 to 25 points, with higher scores denoting a greater capacity for the regulation and management of eating behaviors. In the current investigation, the scale exhibited a Cronbach’s *α* of 0.888, contrasting with the original reported value of 0.750.

#### 2.2.4 E-health literacy

The e-health Literacy Scale (eHEALS) was originally developed in 2006 by Norman et al. (33). The 8-item scale is rated on a 5-point Likert scale ranging from “strongly disagree” (1) to “strongly agree” (5), with higher scores indicating greater perceived e-health literacy. In this study, the scale presents Cronbach’s *α* of 0.932, contrasting with the original reported value of 0.880.

#### 2.2.5 Perceived stress

Perceived stress was assessed using the four-item version of the Perceived Stress Scale (PSS-4), originally derived from the Perceived Stress Scale developed by Cohen et al. (23). The PSS-4 measures the extent to which participants appraised their life as unpredictable, uncontrollable, and overloaded during the past month. Participants rated each item on a 5-point Likert scale ranging from “never” (0) to “very often” (4). Positively worded items were reverse-coded, and all items were summed to generate a total score ranging from 0 to 16, with higher scores indicating higher perceived stress. In the present study, the Cronbach’s *α* of the scale was 0.905.

### 2.3 Statistical methods

Data were analyzed using SPSS 28.0, Mplus 8.3, and PROCESS Macro version 4.0. Descriptive analyses were conducted for all variables. Categorical variables are presented as frequencies and percentages. Non-normally distributed demographic and clinical variables are summarized as medians and interquartile ranges, whereas questionnaire-scale scores in Table 1 are reported as mean ± SD to facilitate comparison of scale distributions and correlation coefficients. Spearman rank correlation analyses were used to examine bivariate associations among eHealth literacy, dietary self-regulation, rest intolerance, and perceived stress. All tests were two-tailed, with *p* < 0.05 indicating statistical significance.

Latent profile analysis was performed in Mplus using the five dietary self-regulation items as continuous profile indicators. Models with one to five profiles were estimated sequentially. To reduce the risk of local maxima, models were estimated using multiple random starts. Model selection was based on AIC, BIC, aBIC, entropy, LMR, BLRT, profile size, and theoretical interpretability. Lower AIC, BIC, and aBIC values, higher entropy, significant LMR and BLRT values, and no very small profile were considered indicators of better model fit. The mean response pattern of each item within each profile was also examined to aid interpretation. The serial mediation analysis was conducted using PROCESS Model 6 in SPSS. eHealth literacy was entered as the independent variable, perceived stress as the first mediator, rest intolerance as the second mediator, and dietary self-regulation as the dependent variable. Gender, age, education level, employment status, residential area, and monthly income were entered as covariates. The total, direct, specific indirect, and serial indirect associations were estimated using 5,000 bootstrap resamples. A 95% bootstrap confidence interval excluding zero was considered statistically significant for indirect associations. Given the cross-sectional design, these results were interpreted as conditional associations rather than evidence of causal mediation or temporal ordering.

LPA was included as a complementary, exploratory person-centered analysis to test whether item-level response patterns contained information beyond the continuous SREBQ total score. It was not intended to establish categorical or stable behavioral subtypes. To examine model dependence, we also estimated the stress–rest intolerance association in three nested linear regression models by adding e-health literacy, then age and gender, and finally the remaining prespecified covariates. The unadjusted comparator was the Spearman correlation reported in Table 1; standardized β coefficients from the nested models are reported in the Results.

## 3 Results

### 3.1 Common method bias test

Harman’s single-factor test yielded four factors with eigenvalues greater than 1, and the first unrotated factor accounted for 44.674% of the total variance. In addition, the VIF values for the four variables were all below 5, indicating no strong multicollinearity among them. Given the limited sensitivity of Harman’s single-factor test as a diagnostic, common method variance cannot be ruled out (34–36). The test was treated only as a sensitivity check and was not used to validate the path model or to justify causal mediation.

### 3.2 Demographic characteristics

The study included 52.0% female and 48.0% male participants, with a median age of 42 years (interquartile range, 33, 59). Most participants were employed (59.6%), lived in urban areas (76.7%), and had a bachelor’s degree (55.9%). The largest proportion reported a monthly household income of ≥9,000 CNY (41.3%), and the median BMI was 21.7 kg/m^2^ (19.0, 24.8). More than 95% of participants had no history of hypertension, diabetes, or hyperlipidemia. Smoking and alcohol consumption were reported by 1.2 and 12.7% of participants, respectively (Table 2).

**Table 2:** Demographic characteristics.

Variable	Category	Frequency	Percentage (%)
Age Median (Q1, Q3)	42 (33, 59)		
Gender	Female	526	52.0%
	Male	485	48.0%
Job	Student	280	27.7%
	Employed	603	59.6%
	Retired	55	5.4%
	Unemployed	73	7.3%
Region	Urban	775	76.7%
	Rural	236	23.3%
Education level	Bachelor’s degree	565	55.9%
	Elementary school	61	6.0%
	High school	89	8.8%
	Junior high school	142	14.0%
	Master’s degree or above	154	15.2%
Average household income per month (yuan)^a^	≤3,000	200	19.8%
	3,000–5,000	184	18.2%
	5,000–9,000	209	20.7%
	>9,000	418	41.3%
BMI, Median (Q1, Q3)	21.7 (19.0, 24.8)		
Waist	≤80	196	19.4%
	81–85	152	15.0%
Waist	86–90	164	16.2%
	91–95	75	7.4%
	>96	59	5.8%
	Unknown	365	36.1%
Hypertension	No	974	96.3%
	Yes	37	3.7%
Diabetes	No	1,001	99.0%
	Yes	10	1.0%
Hyperlipidemia	No	998	98.7%
	Yes	13	1.3%
Smoking	No	961	95.1%
	Yes	12	1.2%
	Quit Smoking	38	3.8%
Alcohol	No	883	87.3%
	Yes	128	12.7%

^a^Yuan is the basic unit of the Renminbi (RMB, the official name of China’s currency).

### 3.3 Associations between dietary self-regulation and e-health literacy, rest intolerance and perceived stress

Spearman correlation analysis revealed that e-health literacy was negatively associated with perceived stress (*r* = −0.508, *p* < 0.01) and positively associated with rest intolerance (r = 0.395, *p* < 0.01) and dietary self-regulation (*r* = 0.482, *p* < 0.01). Perceived stress was negatively correlated with rest intolerance (*r* = −0.146, *p* < 0.01) and dietary self-regulation (*r* = −0.446, *p* < 0.01). In addition, rest intolerance was positively correlated with dietary self-regulation (*r* = 0.569, *p* < 0.01) (Table 1). All correlations reached statistical significance.

**Table 1:** Spearman correlations among main study variables.

Variable	Median (Q1, Q3)	E-health literacy	Perceived stress	Rest intolerance	Dietary self-regulation
E-health literacy	20 (15, 21)	1			
Perceived stress	8 (7, 13)	−0.508**	1		
Rest intolerance	26 (18, 32)	0.395**	−0.146**	1	
Dietary self-regulation	17 (12, 20)	0.482**	−0.446**	0.569**	1

^**^p < 0.01.

### 3.4 Exploratory latent profile analysis

As shown in Table 3 and Figure 2, the AIC, BIC, and aBIC values decreased as the number of classes increased from one to four. Entropy was 0.922, 0.852, and 0.858 for the two-, three-, and four-class solutions, respectively. The LMR test supported the two- and three-class solutions (both *p* < 0.001) but not the four-class solution (*p* = 0.3977); the BLRT was significant for all compared solutions (*p* < 0.001). Considering the fit indices, classification accuracy, class size, and parsimony, the three-class solution was retained. However, the class-specific curves were largely parallel across the five items and differed mainly in overall elevation. The classes are therefore described as low-, moderate-, and high-level groups rather than qualitatively distinct dietary self-regulation subtypes. Comparisons using the low-level class as the reference are presented in Supplementary Table S1. In the retained three-class solution, the estimated profile sizes were approximately 301 participants (29.8%), 197 participants (19.5%), and 512 participants (50.7%). The smallest profile therefore represented nearly one fifth of the sample, supporting the adequacy of the profile sizes for interpretation. Thus, the added value of the LPA in this sample was descriptive: it provided level-based strata and an explicit check for item-level heterogeneity. Because the item-response curves were largely parallel, the analysis did not provide evidence for qualitatively distinct configurations beyond a graded difference in total dietary self-regulation.

**Table 3:** Fit indices for the exploratory latent profile models.

Model	AIC	BIC	ABIC	Entropy	LMR (*p*-value)	BLRT (*p*-value)	Group size
1	16310.081	16359.267	16327.507				
2	13624.660	13703.359	13652.542	0.922	<0.001	<0.001	427.0/584.0
3	13370.188	13478.399	13408.525	0.852	<0.001	<0.001	301.4/197.2/512.4
4	13309.515	13447.239	13358.309	0.858	0.3977	<0.001	260.8/40.1/196.0/514.1

**Figure 2:**
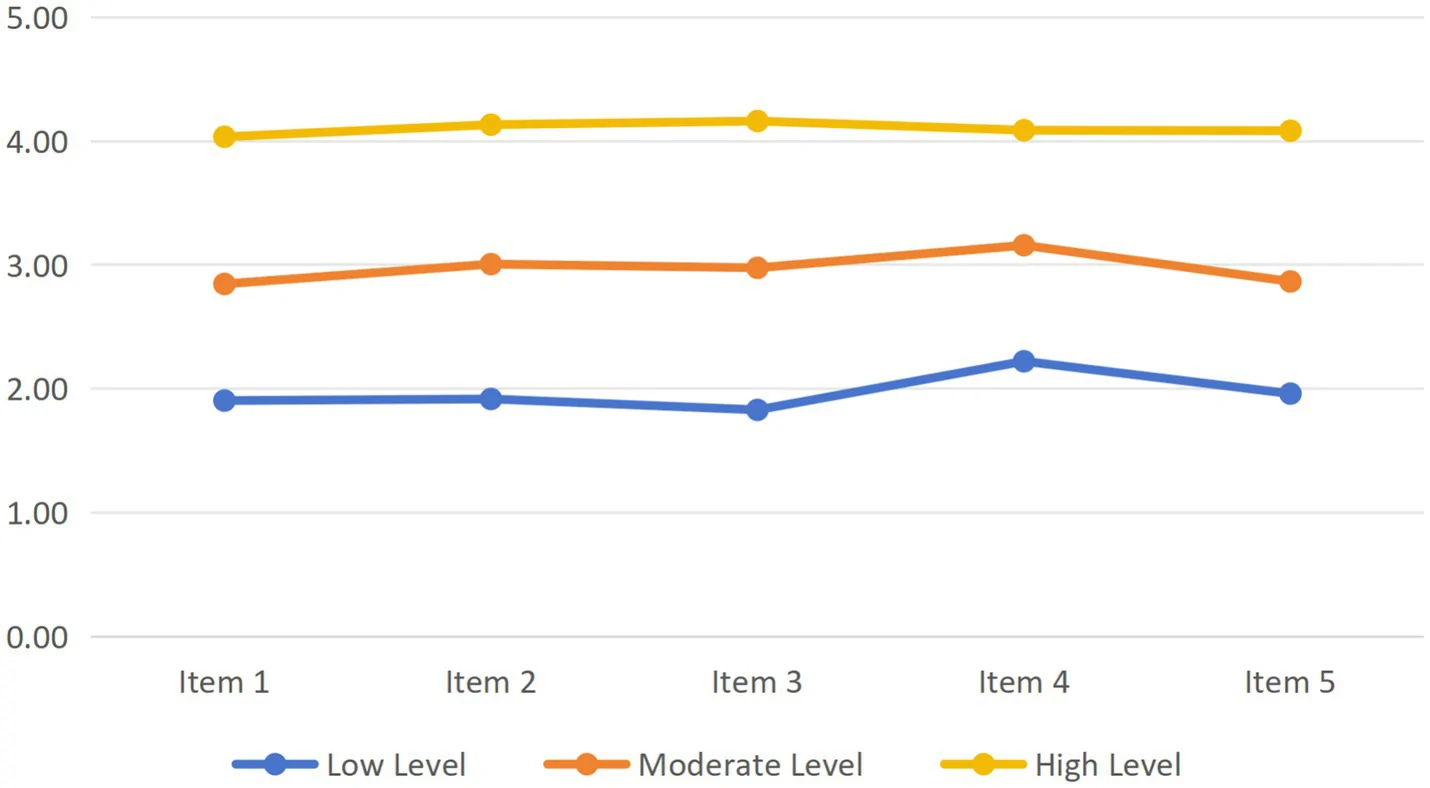
Estimated item means for the three level-based classes of dietary self-regulation. The largely parallel curves indicate primarily quantitative differences in overall level rather than qualitatively different response configurations.

### 3.5 Cross-sectional indirect association analysis

As shown in Table 4, dietary self-regulation was positively associated with e-health literacy (*β* = 0.213, *p* < 0.001) and rest intolerance (*β* = 0.362, *p* < 0.001), and negatively associated with perceived stress (*β* = −0.345, *p* < 0.001). In addition, e-health literacy was negatively associated with perceived stress (*β* = −0.626, *p* < 0.001) and positively associated with rest intolerance (*β* = 0.572, *p* < 0.001). After covariate adjustment, perceived stress showed a weak positive association with rest intolerance (*β* = 0.068, *p* = 0.046), despite their negative bivariate correlation (*r* = −0.146, *p* < 0.01). This direction reversal suggests that the adjusted coefficient was influenced by conditioning on other model variables and should therefore be interpreted with caution. All coefficients represent conditional cross-sectional associations and do not establish causal direction or temporal sequence.

**Table 4:** Main regression coefficients in the prespecified serial indirect association model.

Dependent variable	Predictive variable	*B*	SE	*β*	*t*	*p*
Perceived stress	E-health literacy	−0.519	0.021	−0.626	−24.687	<0.001
Rest intolerance	E-health literacy	0.924	0.056	0.572	16.485	<0.001
Rest intolerance	Perceived stress	0.133	0.066	0.068	1.999	0.046
Dietary self-regulation	E-health literacy	0.211	0.032	0.213	6.526	<0.001
Dietary self-regulation	Perceived stress	−0.413	0.034	−0.345	−12.147	<0.001
Dietary self-regulation	Rest intolerance	0.222	0.016	0.362	13.776	<0.001

*N* = 1,011. The model controls for employment status, education level, age, location, monthly income and gender. *B* refers to the unstandardized regression coefficient, SE stands for standard error, and *β* represents the standardized regression coefficient.

Sensitivity analysis using the final analytical dataset showed that the standardized stress–rest intolerance coefficient changed across staged adjustment: unadjusted bivariate Spearman *r* = −0.146; plus e-health literacy: *β* = 0.097; plus age and gender: *β* = 0.102; fully adjusted: *β* = 0.068. The coefficient therefore changed from a negative bivariate association to a small positive fully adjusted association, supporting indicating model dependence rather than a robust directional relationship.

Based on 5,000 bootstrap resamples, the total association estimate between e-health literacy and dietary self-regulation was 0.615 (95% CI: 0.564–0.666, *p* < 0.001). The association conditional on perceived stress and rest intolerance was 0.211 (95% CI: 0.147–0.274, *p* < 0.001), and the total indirect association estimate was 0.404 (95% CI: 0.344–0.466, *p* < 0.001). The specific indirect association estimate involving perceived stress was 0.214 (95% CI: 0.166–0.261), and that involving rest intolerance was 0.205 (95% CI: 0.161–0.254). The serial indirect estimate was small and negative (estimate = −0.015, 95% CI: −0.031 to −0.001, *p* = 0.048), and its confidence interval only narrowly excluded zero. Given its small magnitude and the direction reversal in the perceived stress–rest intolerance coefficient, this estimate was not treated as evidence of a sequential psychological mechanism (Table 5 and Figure 3).

**Table 5:** Total, conditional, and bootstrap indirect association estimates in the prespecified model.

Association type	Path	Estimate	SE	*t*/*z*	Boot LLCI	Boot ULCI	*p*
Total association	E-health literacy → Dietary self-regulation	0.615	0.026	23.754	0.564	0.666	<0.001
Conditional association	E-health literacy → Dietary self-regulation	0.211	0.032	6.526	0.147	0.274	<0.001
Total indirect association	E-health literacy → Dietary self-regulation	0.404	0.031	13.052	0.344	0.466	<0.001
Specific indirect association 1	E-health literacy → Perceived stress → Dietary self-regulation	0.214	0.024	8.977	0.166	0.261	<0.001
Specific indirect association 2	E-health literacy → Rest intolerance → Dietary self-regulation	0.205	0.024	8.581	0.161	0.254	<0.001
Serial indirect association	E-health literacy → Perceived stress → Rest intolerance → Dietary self-regulation	−0.015	0.008	−1.976	−0.031	−0.001	0.048

Indirect associations were estimated using 5,000 percentile bootstrap resamples. A 95% bootstrap confidence interval excluding zero indicates that the corresponding conditional association estimate is statistically different from zero; it does not establish causal mediation.

**Figure 3:**
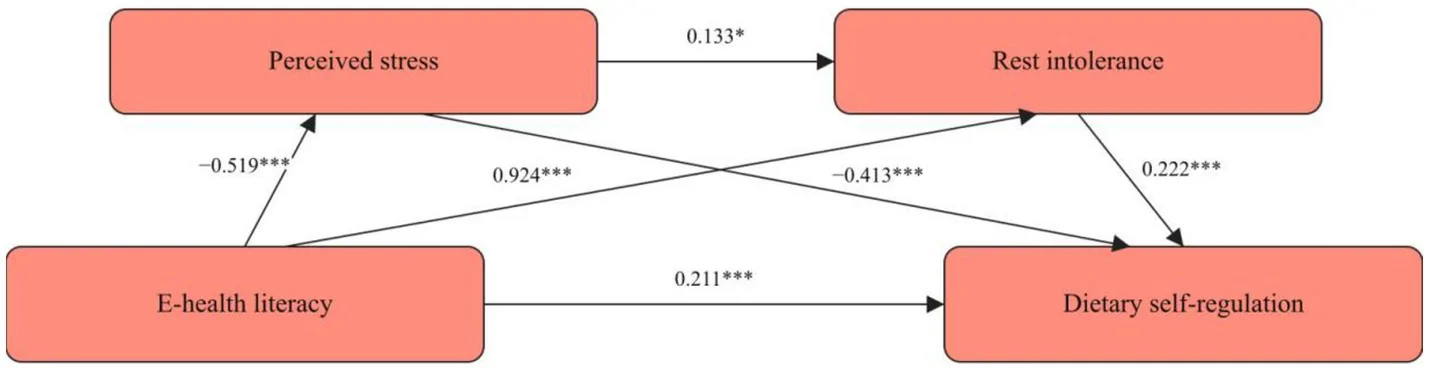
Prespecified cross-sectional path model. Arrows represent fitted regression specifications and do not imply temporal or causal direction. ^***^p < 0.001, ^*^p<0.05.

## 4 Discussion

### 4.1 Summary of main findings

This study yielded two main findings. First, the exploratory latent profile analysis identified three profiles of dietary self-regulation, characterized by low, moderate, and high overall levels. The profiles showed largely parallel item-response patterns, suggesting that participants differed primarily in the degree rather than the type of dietary self-regulation. Thus, although the three-profile solution provided a useful descriptive classification of dietary self-regulation levels in this sample, it did not reveal qualitatively distinct behavioral subtypes.

Second, the cross-sectional path analysis showed that e-health literacy, perceived stress, and rest intolerance were significantly associated with dietary self-regulation. Higher e-health literacy and higher rest intolerance were associated with better dietary self-regulation, whereas higher perceived stress was associated with poorer dietary self-regulation. The model also identified statistically significant specific indirect associations involving perceived stress and rest intolerance. However, the serial indirect association was small and negative, and the association between perceived stress and rest intolerance changed direction after adjustment, indicating that this pathway may be sensitive to model specification. Therefore, the present findings support the presence of cross-sectional covariation among these psychological and behavioral factors, but they do not establish a sequential psychological mechanism.

### 4.2 Level-based variation in dietary self-regulation

The three-class solution indicated that participants differed mainly in the overall level of dietary self-regulation rather than in qualitatively distinct regulatory patterns. Specifically, the five item-response curves were largely parallel across classes, suggesting a graded pattern of low, moderate, and high dietary self-regulation. This finding indicates that, in the present sample, heterogeneity in dietary self-regulation was primarily quantitative rather than typological. Therefore, the three classes should be interpreted as exploratory level-based strata instead of stable behavioral subtypes. This interpretation is consistent with recent methodological recommendations for latent profile analysis, which emphasize that profile solutions should not be treated as substantive subgroups unless they show clear theoretical meaning, external validity, and reproducibility across independent samples (37, 38).

The observed level-based variation in dietary self-regulation is also consistent with recent behavioral nutrition research showing that self-regulatory capacities, including dietary planning, goal monitoring, impulse control, and the ability to maintain dietary intentions in challenging situations, are closely related to healthier eating behaviors (14, 18). Individuals with higher dietary self-regulation are generally more likely to translate nutrition knowledge or health intentions into consistent dietary practices, whereas those with lower self-regulation may be more vulnerable to stress, environmental food cues, and unstable routines (14, 32). In the present study, this pattern was reflected by the ordered distribution of dietary self-regulation scores across the three classes, with the high-level group showing consistently stronger responses across all dietary self-regulation items.

Demographic, socioeconomic, clinical, and lifestyle characteristics also differed across the three level-based classes. These differences suggest that dietary self-regulation may be embedded in broader living conditions rather than reflecting only individual motivation or willpower. Recent evidence indicates that dietary behaviors and self-regulatory capacity are shaped by socioeconomic resources, food accessibility, household context, health status, and daily stress exposure (39, 40). Thus, the group differences observed in this study should be understood as correlates of dietary self-regulation level, not as confirmation of distinct dietary self-regulation subtypes. Before these classes are used to guide targeted interventions, future studies should examine whether the profiles can be replicated longitudinally, predict clinically meaningful dietary or health outcomes, and respond differently to intervention strategies.

### 4.3 Direct association between e-health literacy and dietary self-regulation

E-health literacy was positively associated with dietary self-regulation after adjustment for the prespecified covariates. This pattern is consistent with prior evidence linking e-health literacy to dietary behavior and with transactional models that emphasize access to, understanding of, appraisal of, and application of online health information (41). However, the present data cannot determine whether e-health literacy preceded dietary self-regulation or whether individuals with stronger self-regulation reported greater e-health literacy. Broader evidence links e-health literacy to health-related behaviors, online health-information use, shared decision-making, and wellbeing (42–44). Online nutrition information is frequently inaccurate or of low quality, which supports the importance of e-health and media literacy when evaluating dietary information (45). Intervention studies are needed to determine whether improving e-health literacy produces changes in dietary self-regulation.

### 4.4 Indirect association between perceived stress and dietary self-regulation

The specific indirect association involving perceived stress was 0.214 (95% CI: 0.166–0.261). Specifically, higher e-health literacy was associated with lower perceived stress, and lower perceived stress was further associated with higher dietary self-regulation. This finding is consistent with recent evidence suggesting that individuals with higher digital or e-health literacy are more likely to engage in health-promoting behaviors, obtain and use online health information effectively, and show better self-management capacity (46–48). It is also in line with studies showing that psychological stress is associated with unfavorable eating patterns, emotional eating, poorer dietary control, and obesity-related behavioral processes (22, 24–26, 49). Taken together, these results suggest that perceived stress may be an important psychological correlate linking e-health literacy with dietary self-regulation. However, this indirect association should not be interpreted as causal mediation. Because all variables were measured at the same time point, their temporal order remains unclear, and reverse causality or unmeasured confounding cannot be excluded. Thus, the finding indicates a cross-sectional indirect association consistent with prior evidence, but does not show that e-health literacy reduces perceived stress or improves dietary self-regulation through stress reduction. Longitudinal and intervention studies are needed to test this pathway.

### 4.5 Indirect association between rest intolerance and dietary self-regulation

The specific indirect association estimate involving rest intolerance was 0.205 (95% CI: 0.161–0.254). This positive estimate was unexpected because higher RIS-8 scores indicate stronger negative reactions to rest, rather than greater adaptive tolerance. Therefore, the positive association between rest intolerance and dietary self-regulation should not be interpreted as evidence that rest intolerance enhances self-control or improves eating behavior. In the present analysis, all variance inflation factors were below 5, suggesting that serious multicollinearity among the included variables was unlikely. Thus, the unexpected positive association is unlikely to be explained simply by high collinearity among predictors (50, 51). However, the finding may still reflect residual confounding, response tendencies in self-report measures, unmeasured motivational or health-related factors, or model-specific adjustment effects. Recent methodological studies have emphasized that indirect effects estimated from cross-sectional path models should be interpreted as conditional statistical associations rather than evidence of temporal or causal mechanisms (52–54). Accordingly, although the present study found a statistically non-zero indirect association involving rest intolerance, its behavioral meaning remains uncertain. Further replication, psychometric evaluation of the RIS-8 in community adult samples, and longitudinal or intervention-based studies are needed before this association can be given a substantive behavioral or clinical interpretation. One possibility is that achievement orientation or health consciousness may be positively related to both stronger negative reactions to resting and more deliberate dietary regulation. The association may also reflect common response tendencies because all measures were collected by self-report at the same time. In addition, the RIS-8 was developed and initially evaluated in student samples, so its construct validity and score meaning may differ in a general adult population.

### 4.6 Small serial indirect estimate and directional instability

The prespecified serial indirect estimate involving perceived stress followed by rest intolerance was small and negative (*β* = −0.015, 95% CI: −0.031 to −0.001; *p* = 0.048). Although the confidence interval excluded zero, its interpretation was limited by a direction reversal: perceived stress was weakly negatively correlated with rest intolerance in bivariate analysis but became weakly positive after covariate adjustment. The VIF values for the four variables were all below 5, suggesting no strong multicollinearity; nevertheless, the reversal may reflect statistical suppression, shared variance, or model-specific adjustment effects. Therefore, this pathway should not be interpreted as evidence of a stable psychological mechanism. Overall, the findings indicate cross-sectional covariation among e-health literacy, perceived stress, rest intolerance, and dietary self-regulation, but alternative temporal orderings, residual confounding, and common method variance remain possible. Longitudinal or experimental studies are needed to test directionality and causal processes.

### 4.7 Implications for community-based nutrition education and health promotion

The findings also suggest several hypotheses for future community-based nutrition education and health-promotion research. Consistent with socio-ecological and COM-B perspectives (6, 7), future programs delivered through community health centers could combine practical digital health-literacy training with stress-management support and concrete dietary self-regulation skills, such as goal setting, meal planning, self-monitoring, and evaluation of online nutrition information. Because rest intolerance includes guilt, persistent task-related thinking, and difficulty disengaging during rest, intervention studies could also examine whether helping participants recognize and manage these reactions improves engagement with dietary education. Program content may need to be adapted for age, baseline e-health literacy, and access to digital resources. Importantly, the present cross-sectional associations do not justify causal or clinical recommendations. These possibilities should be tested in longitudinal studies or community-based randomized trials that evaluate dietary behavior, psychological outcomes, implementation feasibility, and sustainability over time.

## 5 Limitations

This study has several limitations. First, although age-quota matching was used, participants were recruited through convenience sampling and the final sample included only permanent residents of Shanghai; representativeness across characteristics other than age and generalizability to other populations may therefore be limited. Second, the cross-sectional design precludes conclusions about temporal order or causal mediation; the direct and indirect effects should be interpreted as conditional associations within the specified model. Third, all key variables were collected by self-report at one time point, so recall bias and common method variance may be present. Fourth, the three latent profiles were based on five dietary self-regulation items and showed largely parallel patterns, suggesting low, moderate, and high overall levels rather than validated qualitative subtypes. Fifth, the positive association between rest intolerance and dietary self-regulation was unexpected, and the direction reversal between bivariate and adjusted stress–rest intolerance associations suggests possible suppression or model dependence. Finally, the serial indirect estimate was small and should be replicated in longitudinal studies with further measurement validation.

The 234 questionnaires removed during quota matching were not a random subsample, and age matching alone cannot correct for selection on education, health status, internet access, or health-related interest. The high proportion of participants with a bachelor’s degree or higher and the very low reported prevalence of several chronic conditions and smoking further suggest that the sample may be healthier and more highly educated than the wider Shanghai adult population. These features limit external validity and should be considered when interpreting the findings or planning community interventions.

## 6 Conclusion

This study identified three latent profiles of dietary self-regulation among community adults, reflecting low, moderate, and high overall levels rather than qualitatively distinct behavioral subtypes. The path analysis showed that e-health literacy, perceived stress, and rest intolerance were significantly associated with dietary self-regulation. Higher e-health literacy and lower perceived stress were related to better dietary self-regulation, while the positive association involving rest intolerance was unexpected and should be interpreted cautiously. Although the VIF values of the four variables were all below 5, suggesting no strong multicollinearity, the direction reversal between perceived stress and rest intolerance after adjustment indicates that some coefficients may be model-dependent. Overall, the findings describe cross-sectional covariation among e-health literacy, perceived stress, rest-related negative responses, and dietary self-regulation, but they do not establish causal direction or a sequential mediation mechanism. Future studies using longitudinal designs, experimental interventions, and further psychometric validation of the RIS-8 are needed to clarify temporal relationships and determine whether these factors can be targeted to improve dietary self-regulation. Community-based intervention studies should examine whether integrated support for e-health literacy, stress management, and dietary self-regulation can improve dietary behavior.

## Data Availability

The datasets presented in this study can be found in online repositories. The names of the repository/repositories and accession number(s) can be found below: https://www.x-mol.com/groups/pbicr/research.
