# Supplementary material for: Dietary self-regulation levels among adults in Shanghai: cross-sectional associations with e-health literacy, perceived stress, and rest intolerance
Source: Front Public Health. 2026 Oct 27;14:1928922. doi: 10.3389/fpubh.2026.1928922 (PMC13760829; doi:10.3389/fpubh.2026.1928922)
Supplement: Supplementary file 1 [file Table_1.DOCX]

**Supplementary Table 1 Multivariate analysis between Low, Moderate and High Level**

| **Variable** | **Low** | **Moderate** | **OR (95% CI)** | **p** | **Variable** | **Low** | **High** | **OR (95% CI)** | **p** |
| --- | --- | --- | --- | --- | --- | --- | --- | --- | --- |
| **Age** | 308.00 | 183.00 | 1.704 (1.297, 2.239) | <0.001*** | **Age** | 308.00 | 520.00 | 0.997 (0.987, 1.008) | 0.63 |
| **Gender** |  |  |  |  | **Gender** |  |  |  |  |
| Female | 165.00 | 87.00 | — |  | Female | 165.00 | 274.00 | — |  |
| Male | 143.00 | 96.00 | 0.74 (0.471, 1.165) | 0.19 | Male | 143.00 | 246.00 | 1.001 (0.742, 1.352) | 0.99 |
| **Region** |  |  |  |  | **Region** |  |  |  |  |
| City | 235.00 | 166.00 | — |  | City | 235.00 | 374.00 | — |  |
| Countryside | 73.00 | 17.00 | 1.427 (0.746, 2.730) | 0.28 | Countryside | 73.00 | 146.00 | 0.835 (0.591, 1.179) | 0.31 |
| **Education level** |  |  |  |  | **Education level** |  |  |  |  |
| Elementary school | 21.00 | 4.00 | — |  | Elementary school | 21.00 | 36.00 | — |  |
| High school | 20.00 | 20.00 | 0.342 (0.082, 1.421) | 0.14 | High school | 20.00 | 49.00 | 0.642 (0.296, 1.391) | 0.26 |
| Junior high school | 44.00 | 15.00 | 0.558 (0.139, 2.235) | 0.41 | Junior high school | 44.00 | 83.00 | 0.948 (0.484, 1.856) | 0.88 |
| Bachelor's degree | 181.00 | 116.00 | 0.400 (0.113, 1.419) | 0.16 | Bachelor's degree | 181.00 | 268.00 | 1.056 (0.584, 1.907) | 0.86 |
| Master's degree or above | 42.00 | 28.00 | 0.379 (0.097, 1.477) | 0.16 | Master's degree or above | 42.00 | 84.00 | 0.725 (0.366, 1.434) | 0.36 |
| **Job** |  |  |  |  | **Job** |  |  |  |  |
| Employed | 191.00 | 122.00 | — |  | Employed | 191.00 | 290.00 | — |  |
| Student | 82.00 | 48.00 | 1.350 (0.816, 2.232) | 0.24 | Student | 82.00 | 150.00 | 0.849 (0.604, 1.195) | 0.35 |
| Retired | 6.00 | 12.00 | 0.213 (0.069, 0.658) | 0.007** | Retired | 6.00 | 37.00 | 0.251 (0.097, 0.652) | 0.005** |
| Unemployed | 29.00 | 1.00 | 15.535 (1.967, 122.715) | 0.009** | Unemployed | 29.00 | 43.00 | 1.113 (0.657, 1.886) | 0.69 |
| **Average household income per**  **month (yuan)** |  |  |  |  | **Average household income per**  **month (yuan)** |  |  |  |  |
| ≤3000 | 61.00 | 13.00 | — |  | ≤3000 | 61.00 | 126.00 | — |  |

**Continue**

| **Variable** | **Low** | **Moderate** | **OR (95% CI)** | **p** | **Variable** | **Low** | **High** | **OR (95% CI)** | **p** |
| --- | --- | --- | --- | --- | --- | --- | --- | --- | --- |
| 3000-5000 | 53.00 | 29.00 | 0.554 (0.243, 1.262) | 0.16 | 3000-5000 | 53.00 | 102.00 | 1.104 (0.692, 1.762) | 0.68 |
| 5000-9000 | 70.00 | 50.00 | 0.578 (0.267, 1.247) | 0.16 | 5000-9000 | 70.00 | 89.00 | 1.561 (0.990, 2.463) | 0.06 |
| ＞9000 | 124.00 | 91.00 | 0.539 (0.262, 1.111) | 0.09 | ＞9000 | 124.00 | 203.00 | 1.22 (0.816, 1.823) | 0.33 |
| **BMI** | 308.00 | 183.00 | 0.902 (0.722, 1.128) | 0.37 | **BMI** | 308.00 | 520.00 | 1.007 (0.975, 1.040) | 0.66 |
| **Waist** |  |  |  |  | **Waist** |  |  |  |  |
| ≤80 | 62.00 | 49.00 | — |  | ≤80 | 62.00 | 85.00 | — |  |
| 81-85 | 38.00 | 37.00 | 0.736 (0.370, 1.464) | 0.38 | 81-85 | 38.00 | 77.00 | 0.694 (0.409, 1.176) | 0.18 |
| 86-90 | 49.00 | 18.00 | 1.707 (0.793, 3.676) | 0.17 | 86-90 | 49.00 | 97.00 | 0.701 (0.424, 1.161) | 0.17 |
| 91-95 | 23.00 | 8.00 | 1.269 (0.460, 3.504) | 0.65 | 91-95 | 23.00 | 44.00 | 0.759 (0.401, 1.437) | 0.40 |
| ≥96 | 19.00 | 4.00 | 2.591 (0.675, 9.946) | 0.17 | ≥96 | 19.00 | 36.00 | 0.783 (0.398, 1.540) | 0.48 |
| Unknown | 117.00 | 67.00 | 1.149 (0.658, 2.006) | 0.63 | Unknown | 117.00 | 181.00 | 0.939 (0.613, 1.438) | 0.77 |
| **Hypertension** |  |  |  |  | **Hypertension** |  |  |  |  |
| Yes | 7.00 | 15.00 | — |  | Yes | 7.00 | 15.00 | — |  |
| No | 301.00 | 168.00 | 2.016 (0.616, 6.601) | 0.25 | No | 301.00 | 505.00 | 1.017 (0.325, 3.180) | 0.98 |
| **Diabetes** |  |  |  |  | **Diabetes** |  |  |  |  |
| Yes | 0.00 | 6.00 | — |  | Yes | 0.00 | 4.00 | — |  |
| No | 308.00 | 177.00 | 4.3 99 ×10^6^ (0.000, Inf) | 0.98 | No | 308.00 | 516.00 | 1.171 ×106 (0.000, Inf) | 0.97 |
| **Hyperlipidemia** |  |  |  |  | **Hyperlipidemia** |  |  |  |  |
| Yes | 6.00 | 5.00 | — |  | Yes | 6.00 | 2.00 | — |  |
| No | 302.00 | 178.00 | 0.653 (0.139, 3.061) | 0.59 | No | 302.00 | 518.00 | 0.093 (0.010, 0.885) | 0.039* |
| **Smoking** |  |  |  |  | **Smoking** |  |  |  |  |
| Yes | 9.00 | 18.00 | — |  | Yes | 9.00 | 11.00 | — |  |

**Continue**

| **Variable** | **Low** | **Moderate** | **OR (95% CI)** | **p** | **Variable** | **Low** | **High** | **OR (95% CI)** | **p** |
| --- | --- | --- | --- | --- | --- | --- | --- | --- | --- |
| No | 297.00 | 158.00 | 2.349 (0.898, 6.144) | 0.08 | No | 297.00 | 506.00 | 0.889 (0.328, 2.406) | 0.82 |
| Quit Smoking | 2.00 | 7.00 | 0.984 (0.149, 6.481) | 0.99 | Quit Smoking | 2.00 | 3.00 | 1.877 (0.184, 19.119) | 0.60 |
